# Interactions between host and gut microbiota in gestational diabetes mellitus and their impacts on offspring

**DOI:** 10.1186/s12866-024-03255-y

**Published:** 2024-05-10

**Authors:** Shuxian Wang, Zifeng Cui, Huixia Yang

**Affiliations:** 1 Department of Obstetrics and Gynaecology, Peking University First Hospital, Beijing, China; 2 Beijing Key Laboratory of Maternal Fetal Medicine of Gestational Diabetes Mellitus, Beijing, China

**Keywords:** Gut microbiota, Gestational diabetes mellitus (GDM), Host-microbiome interactions, Metabolism, Offspring

## Abstract

Gestational diabetes mellitus (GDM) is characterized by insulin resistance and low-grade inflammation, and most studies have demonstrated gut dysbiosis in GDM pregnancies. Overall, they were manifested as a reduction in microbiome diversity and richness, depleted short chain fatty acid (SCFA)-producing genera and a dominant of Gram-negative pathogens releasing lipopolysaccharide (LPS). The SCFAs functioned as energy substance or signaling molecules to interact with host locally and beyond the gut. LPS contributed to pathophysiology of diseases through activating Toll-like receptor 4 (TLR4) and involved in inflammatory responses. The gut microbiome dysbiosis was not only closely related with GDM, it was also vital to fetal health through vertical transmission. In this review, we summarized gut microbiota signature in GDM pregnancies of each trimester, and presented a brief introduction of microbiome derived SCFAs. We then discussed mechanisms of microbiome-host interactions in the physiopathology of GDM and associated metabolic disorders. Finally, we compared offspring microbiota composition from GDM with that from normal pregnancies, and described the possible mechanism.

## Introduction

During normal pregnancy, women undergo diverse physiological adaptations including increased insulin resistance (IR) [1]. In susceptible populations who are incapable of producing enough insulin, GDM occurred [2]. Besides the short and long-term adverse effects such as fetal macrosomia and type 2 diabetes (T2DM), children born to GDM mothers are at increased risk for obesity and metabolic syndrome later in life [3]. Gut microbiome is involved in body weight control, energy homeostasis and absorption of no-digestible fiber. Previous studies showed particularities of microbiome’s action during normal and GDM pregnancies [4, 5]. The common findings were reduction in short chain fatty acid (SCFA)-producing genera and dominant of Gram-negative pathogens releasing lipopolysaccharide (LPS) [6, 7]. SCFAs are closely related with maternal-fetal glycolipid parameters [8]. They functioned as energy sources for colonocytes, owned potential capacity to modulate immune-metabolic responses or gut barrier integrity, and regulated insulin secretion through promoting GLP-1 and PYY secretion [9]. They were also absorbed into the circulation and engaged specific receptors to activate downstream signaling pathways that ultimately impacted cellular processes. LPS was a bacterial endotoxin which destroyed gut mucosal barrier by activating TLR4 or decreasing SCFAs, and involved in metabolic inflammation [10]. The microbiome could be transmitted to fetus and determined early microbial composition [11]. In this review, we summarized current evidence regarding gut microbiota signature in GDM pregnancies. We then presented a brief introduction of SCFAs and discussed mechanisms of microbiome-host interactions in the physiopathology of GDM and associated metabolic disorders. Finally, we compared neonatal microbiota composition between GDM and normal pregnancies, and described the influential pathways.

## Gut microbiota profile in pregnancies complicated by GDM

**Fig. 1:**
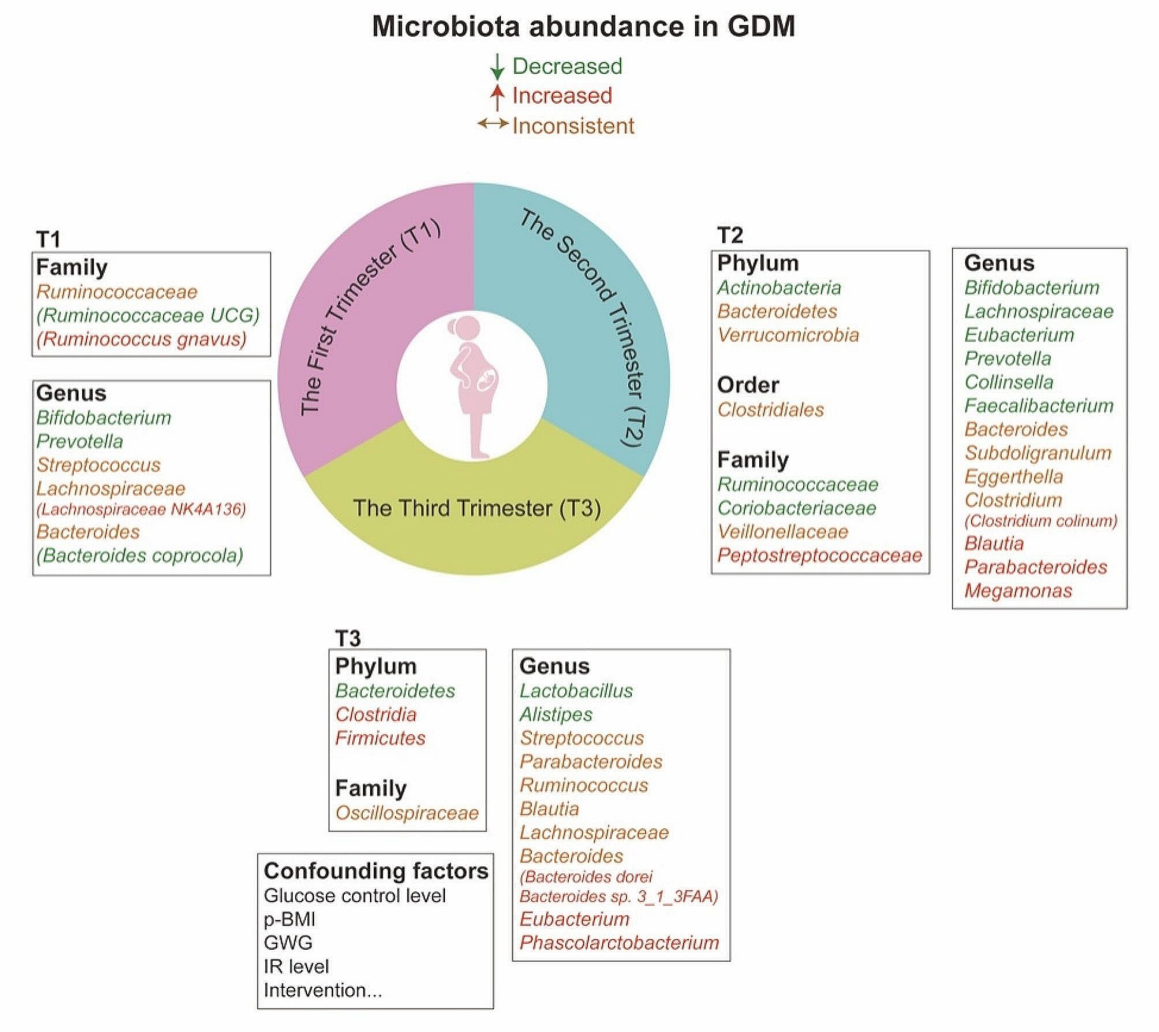
Gut microbiota profile in pregnancies complicated by GDM during each trimester. In GDM pregnancies during each trimester, the microbiota with decreased abundance were shown in green, and the opposite were shown in red. The distribution of some microbiome has not been consistently concluded and were shown in orange

The microbiome profiles of GDM were summarized in Table 1; Fig. 1 during each trimester [4, 6, 7, 12–38]. Totally, GDM was mostly linked to an elevated *Firmicutes/Bacteroidetes* (F/B) ratio, decreased richness and diversity which was associated with enhanced capacity to harvest energy and akin to that found in obesity [12–15]. Microbiome in early pregnancy was considered as diagnostic marker for GDM [16–18]. The genera *Eisenbergiella* and *Tyzzerella 4* were upregulated and positively correlated with fasting plasma glucose (FPG) among pregnancies diagnosed with GDM subsequently [19]. Normoglycemic women were abundant in family *Prevotellaceae*, order *Fusobacteriales*, and genus *Subdoligranulum*, which was negatively correlated with LDL levels [20]. The differences were negligible in another randomized controlled trial (RCT), which was due to different technology platform, demographic characteristics and diagnostic criteria [13]. It is debated that intestinal microbiota is a cause or consequence of GDM, and fecal microbiota transfer (FMT) experiments are needed. GDM-recipient mice obtained differential microbial communities demonstrated as reduced *P.copri* and increased IL-6 level [21].The GDM prediction model containing clinical information, microbial and inflammatory markers obtained high accuracy [21]. Although gut microbiome dysbiosis could be the first response to GDM onset, the phenotype transfer may also be caused by metabolites and eukaryotic microorganisms. It is meaningful to further unravel the underlying mechanisms in terms of these fecal derived material [21].

**Table 1:** Clinical characteristics of included studies and alterations of gut microbiome in pregnancies complicated by GDM

Region	Year	Participants	Sampling time	Sequencing methods	GDM associated microbiota		Ref.
					Decreased	Increased	
Brazil	2019	GDM: 26 Women without GDM: 42 Nutrient intake evaluation (-)	T3: 28-36w	16 S rRNA V4	*Bacteroidetes: Bacteroides Parabacteroides*, *Roseburia, Dialister, Akkermansia*	*Firmicutes: Ruminococcus, Eubacterium, Prevotella*, *Lachnospiraceae, Phascolarctobacterium*, *Christensenellaceae*,	[4]
China	2019	GDM: 23 Women without GDM: 26 Nutrient intake evaluation (-)	T3	Metagenomics	*Alistipes putredinis, Lactobacilluscasei*	*Bacteroides: Bacteroides dorei, Bacteroides sp. 3_1_3FAA*	[6]
China	2021	GDM late pregnancy: 27 Health late pregnancy: 30 Health early pregnancy: 50 Nutrient intake evaluation (-)	T1 T3	16 S rRNA V3-V4	*Lactobacillus, Bifidobacterium*,	*Escherichia, Streptococcus, Proteobacteria*	[7]
China	2021	GDM: 110 Healthy women: 220 Nutrient intake evaluation (-)	T2: 22-24w Before GDM diagnosis	16 S rRNA V3-V4	*Firmicutes: Veillonellaceae, Lachnospiraceae, Ruminococcaceae, Oscillospira, Ruminococcaceae;g, Clostridiales, Gemmiger* *Actinobacteria: Bifidobacterium, Coriobacteriaceae*	*Bacteroidetes: Bacteroides, Rikenellaceae, Butyricimonas, Odoribacter*	[12]
Finland	2021	Overweight/Obesity GDM: 67 Early onset: 14 Mid-pregnancy onset: 53 Women without GDM: 203 Nutrient intake evaluation (-)	Early pregnancy:13.9w Late pregnancy: 35.2w	Metagenomics	*No differences in gut microbiota between women with and without GDM* *Increased Megasphaera in early pregnancy among confirmed onset GDM;* *Increased F/B ratio with early onset GDM in late pregnancy;* *Increased R.obeum, S. wadsworthensis, Subdoligralunum in mid pregnancy onset GDM in fish oil + probiotics group;*		[13]
Danmark	2018	GDM: 50 Healthy control: 157 Nutrient intake evaluation (+)	T3: 27-33w 8 months postpartum	16 S rRNA V1-V2	*14 OTUs assigned to Acetivibrio, Intestinimonas, Erysipelotrichaceae incertae sedis, Isobaculum, Butyricicoccus, Clostridium IV/XVIII, Oscillibacter, Ruminococcus, Bacteroides, Veillonella, Suterella*	*Three OTUs assigned to Blautia, Ruminococcus, Faecalibacterium*	[14]
Thailand	2021	GDM unsuccessful diet control (GDM-U): 13 GDM successful diet control (GDM-S): 28 Non-GDM: 38 Nutrient intake evaluation (+)	T2: 24-28w T3: before delivery Newborns	16 S rRNA	T2: GDM-U: *Lactobacillales, Bacteroidetes*, T2/T3: GDM-U: *Eubacteria, Enterobacteriaceae* T2: GDM-S: *Lactobacillales.*	T2/T3: GDM-U: F/B ratio T3: GDM-S: F/B ratio	[15]
China	2020	GDM: 31 Healthy control: 103 Nutrient intake evaluation (-)	T1: 8-12w T2: 24-28w	16 S rRNA V3-V4	*T1/T2: Flavonifractor, Streptococcus, Coprococcus* *Megasphaera, Eggerthella* *T1: Prevotella, Coprococcus, Streptococcus, Peptococcus, Desulfovibrio*, *Intestinimonas, Veillonella.*	*T2: Holdemania, Megasphaera, Eggerthella*	[16]
China	2021	GDM: 201 Control: 201 Nutrient intake evaluation (+)	T1: 6-15w	16 S rRNA V3-V4	*Cyanobacteria*, *Actinomyces: Rothia,, Actinobacteria, Bifidobacterium Adlercreutzia, Coriobacteriaceae, Lachnospiraceae spp.*	*Enterobacteriaceae, Ruminococcaceae spp., Veillonellaceae, Proteobacteria*,	[17]
China	2023	GDM: 120 Control: 120	T1: <15 + 6w T2: 24-28w T3: >29w	Metagenomics	T1-T3: *Ruminococcus bromii* T1-T2: *Alistipes putredinis and Bacteroides ovatus*	T1-T3: 10 GDM-related species *(e.g., Alistipes putredinis)*	[18]
China	2020	GDM: 98 Health control: 98 Nutrient intake evaluation (+)	T1: 10-15w	16 S rRNA V4	*Parabacteroides, Megasphaera, Eubacterium eligens group*, *Parasutterella, Dialister, Ruminococcaceae UCG 005/002/003, Eubacterium xylanophilum group*	*Eisenbergiella, Tyzzerella 4, Lachnospiraceae NK4A136*	[19]
Czechia	2022	Healthy control: 22 GDM1: 29 (impaired FPG in T1) GDM2:31 (impaired FPG in T3) GDM3: 22 (impaired OGTT in T3) Nutrient intake evaluation (-)	T1 T3	16 S rRNA V3-V4	T1: Family: *Prevotellaceae*, Order: *Fusobacteriales*, Genus: *Sutterella;* Class: *Bacteroidia, g-Proteobacteria*, T3: Class: *Desulfovibrionea, Bacilli;* Genus: *Bilophila, Leuconostoc, Streptococcus, Erysipelotrichaceae UCG-003*	T1: Genus: *Enterococcus, Erysipelotrichaceae UCG-003;* T3: Class: *Negativicutes, Clostridia*, Family: *Oscillospiraceae, Debaryomycetaceae* Genus: *Rhodotorula*	[20]
Israel	2023	GDM: 44 Control: 350 Nutrient intake evaluation (+)	T1:11 + 0–13/-6w	16 S rRNA V4	*Prevotella* and other 16 bacteria; 15 species after controlling for age and BMI (6 were intersected)	1 bacteria species	[21]
China	2021	GDM: 30 Health pregnant women: 28 Nutrient intake evaluation (-)	T2: 24-28w	16 S rRNA	*Bacteroides spp., Bacillus spp., Bifidobacterium spp., Clostridium spp., Eubacterium spp., Prevotella* *spp.*	*Corynebacterium spp, Lactobacillus* *spp., Blautia hydrogenotrophica*	[22]
China	2021	GDM: 21 Normoglycemic women: 32 Nutrient intake evaluation (+)	T2: 24-28w	16 S rRNA V3-V4	*Proteobacteria, Actinobacteria, Verrucomicrobia, Tenericutes* Genus: *Escherichia shigella, Ruminococcaceae UCG014 Eubacterium coprostanoligenes group, Christensenellaceae R7 group, Subdoligranulum, Akkermansia, Collinsella, Lachnospiraceae UCG004, Rhodococcus, Desulfovibrio*	*Bacteroidetes* Genus: *Incertaesedis, Citrobacter, Parabacteroides, Fusicatenibacter*	[23]
China	2021	GDM: 15 Normal glucose tolerance: 18 Nutrient intake evaluation (-)	T2: 24-28w	16 S rRNA V4 qPCR	*Bacteroidetes, Bacteroidia, Bacteroidales*	*Clostridiales, Clostridia, Firmicutes; Ruminococcus bromii, Clostridium colinum, Streptococcus infantis*	[24]
China	2022	GDM: 50 Normal glucose tolerance (NGT): 54	T2: 24-28w	Metagenomics	Genus: *Faecalibacterium, Prevotella*, and *Streptococcus* *Prevotella/Bacteroides* ratio, Species: *Bacteroides coprophilus, Eubacterium siraeum, Faecalibacterium prausnitzii, Prevotella copri*, and *Prevotella stercorea*	Phylum: *Verrucomicrobia*, Genus: *Megamonas.* Species: *Bacteroides eggerthii, Megamonas unclassified, Ruminococcus gnavus*	[25]
China	2017	GDM: 43 Normal: 81 Nutrient intake evaluation (-)	21-29w	Metagenomics	Order: *Clostridiales*, Family: *Coriobacteriaceae* Genus: *Ruminiclostridium, Roseburia, Eggerthella, Fusobacterium*, *Haemophilus, Mitsukella, Aggregatibacter*	Genus: *Parabacteroides, Megamonas, Phascolarctobacterium*	[26]
China	2019	GDM1: successful glycemic control: 24 GDM2: failure of glycemic control: 12 Normal: 16 Nutrient intake evaluation (+)	T2: 24-28w	16 S rRNA V3-V4	*Faecalibacterium*, *Subdoligranulum* (GDM2), *Phascolarctobacterium* (GDM2) *Roseburia* Compared with N and GDM1: *Faecalibacterium, Subdoligranulum*	*Blautia, Eubacterium_hallii_group*, Compared with N and GDM1: *Blautia, Eubacterium_hallii_group*	[27]
China	2022	GDM with medical nutrition therapy (MNT) Effective group: 62 Ineffective group: 12	T2: 24-28w	16 S rRNA V4	Before treatment: Ineffective group was enriched in *Desulfovibrio, Aeromonadales, Leuconostocaceae, Weissella, Prevotella, Bacillales_Incertae Sedis XI, Gemella* and *Bacillales;* Effective group was enriched in *Roseburia, Clostridium, Bifidobacterium, Bifidobacteriales, Bifidobacteriaceae, Holdemania* and *Proteus.* After treatment: Effective group was enriched in *Bifidobacterium and Actinomycete*, Ineffective group was enriched in *Holdemania, Proteus, Carnobacteriaceae and Granulicatella.*		[28]
China	2023	GDM: 14 Control: 41 (23 normal pregnancies)	T1 T2 T3	Metagenomics	T1-T3: *Bacteroides coprocola, Bacteroides plebeius, Erysipelatoclostridium ramosum*, and *Prevotella copri*	T1-T3: *Ruminococcus_gnavus, Akkermansia_muciniphila, Alistipes_shahii*, *Blautia_obeum*, and *Roseburia_intestinalis*	[29]
China	2020	GDM: 20 Non-diabetes control: 29 Nutrient intake evaluation (-)	Medium: 34w	16 S rRNA V3-V4 1 H-NMR	*Blautia*	*Phascolarctobacterium, Alistipes, Parabacteroides, Eubacterium coprostanoligenes, Oscillibacter, Paraprevotella, Ruminococcaceae NK4A214*	[30]
Australia	2021	Overweight/Obesity GDM: 29 Euglycaemic women: 29 Nutrient intake evaluation (+)	T2: 16w T3: 28w	16 S rRNA V6-V8	T3: *Phylum Bacteroidales, Family Lachnospiraceae, Genera Lachnospira*	T3: *Genus Blautia*	[31]
China	2020	GDM: 30 Normal control: 31 Nutrient intake evaluation (-)	T3	16 S rRNA V3-V4	*Rikenellaceae, Alistipes, Phascolarctobacterium*	Class *Gammaproteobacteria*, Genus *Hemophilus, Pasteurellaceae*	[32]
China	2021	GDM: 7 Periodontitis: 28 GDM + Periodontitis: 7 Normal control: 27 Nutrient intake evaluation (-)	T2: 20-28w	16 S rRNA V4	*s_Lactococcus_lactis, Desulfobacteraceae*	GDM: *f_Lachospiraceae, Defluviitaleaceae, Paracaedibacteraceae* GDM + Periodontitis: *s-bacterium_enrichment_culture_clone_R4-81B, Methanobacteriales, Nostocaceae*	[33]
China	2021	GDM: 23 (LG: *n* = 12; G: *n* = 11) Normoglycemic women: 29 Nutrient intake evaluation (-)	T3: after 28w	16 S rRNA V3-V4	*Bacteroides, Bacteroidetes*, *Bacteroidales, Bacteroidia, Betaproteobacteria*, *Alcaligenaceae, Sutterella, Burkholderiales, Pyramidobacter*, *Dethiosulfovibrionacea.*	*Firmicutes, Coriobacteriaceae, Coriobacteriia, Coriobacteriales, Collinsella, Dorea, Coprococcus, Ruminococcus, Ruminococcaceae*, *Lachnospira, Blautia, Lachnospiraceae, Clostridiales*, *Clostridia*	[34]
China	2020	GDM: 59 Health control: 48 Nutrient intake evaluation (-)	T2: 24-28w	16 S rRNA V3-V4	Family: *Enterobacteriaceae, Ruminococcaceae,, Lachnospiraceae*	Family: *Peptostreptococcaceae, Veillonellaceae, Erysipelotrichaceae, Prevotellaceae, Verrucomicrobiaceae*,	[35]
Italy	2018	GDM: 41 Nutrient intake evaluation (+)	T2: 24-28w T3: 38w	16 S rRNA V3-V4	GDM adherents: *Bacteroides* decreased GDM non-adherents: *Faecalibacterium and L-Ruminococcus increased*		[36]
Japan	2021	GDM: 20 NGT: 16 Nutrient intake evaluation (-)	T2: GDM diagnosis T3: 35-37w 4weeks postpartum	16 S rRNA	Compared with non-OW/OB GDM-T2: *Collinsella;* T3/Postpartum: *Ruminococcus* Postpartum: *Prevotella 9*	T2/T3: *Peptostreptococcaceae, Romboutsia* non-OW/OB GDM: *Verrucomicrobia, Coriobacteriaceae, Akkermansiaceae* in T3	[37]
Brazil	2022	Overweight or Obese GDM: 36(T1/T2)54 (T3) Control: 54 (T1/T2) 55 (T3) Nutrient intake evaluation (+)	T1 T2 T3	16 S rRNA V4	-	Genus: *Bacteroides*	[38]

T1: The first trimester, T2: The second trimester, T3: The third trimester

Most studies proposed a specific microbiome composition at time of GDM diagnosis. The genera *Bifidobacterium*, *Prevotella*, and *Ruminococcaceae UCG014* were significantly decreased, while *Parabacteroides* and *Blautia* were increased in GDM [22–24]. Most of the reduced flora were SCFA-producing genera and positively contributed to dopaminergic synapse, betalain biosynthesis, and isoquinoline alkaloid biosynthesis. They were also negatively correlated with glucose level, visceral fat area and inflammatory cytokines in single and co-occurrence network analysis, reflecting the microbe interactions within an ecosystem [25]. In pregnant women with impaired FPG, valerate and 2-hydroxybutyrate were positively related with archaeon *Methanobrevibacter* and genus *Phascolarctobacterium*, and became prognostic markers for diabetes related complications [20]. Metagenome linkage groups (MLGs) were clustered from co-abundance genes physically linked rather than independently distributed by metagenomics analysis. GDM was enriched with MLGs of *Enterobacteriaceae* and positively correlated with glucose level [26]. Transferring fecal microbiota from GDM to germ-free (GM) mice induced hyperglycemia and decreased SCFA-producing *Akkermansia* [39]. It supported the multiple parallel hits that gut microbiome dysbiosis primed SCFA imbalance and metabolic inflammation, and contributed to GDM development [25]. The efficiency of glucose control level contributed to the inconsistent findings demonstrated as upregulated [12, 23] or downregulated [22, 24] genus *Bacteroides* in GDM. The microbiome composition of women with successful glycemic control was more similar with those from normal pregnancies, and harbored a unique microbiome pattern abundance of genera *Roseburia*, *Clostridium*, and *Bifidobacterium* [27, 28]. They were negatively correlated with blood glucose level and help correct GDM conditions.

GDM status was the main factor that affected microbiome changes at late pregnancy [29]. Compared with normal pregnancies, time-dependent alterations of F/B ratio and 𝛼-diversity were not observed in GDM [18]. Genera *Bifidobacterium* and *Ruminococcaceae* were still decreased and lasted to postpartum [7, 14]. There are some cofactors that cannot be ignored given that previous upregulated *Blautia* and decreased *Eubacterium* were found to be inversed [30]. A composite microbial risk score (CMRS) was calculated based on ten GDM-related species. Its association with glycemic traits was significantly modified by habitual intake of fiber-rich plant foods [18]. In GDM with unsuccessful diet control, the genera *Eubacteria* and *Enterobacteriaceae* were significantly lower, and F/B ratio was higher before delivery [15]. GDM pregnancies adherent to nutritional recommendations demonstrated obvious decrease in *Bacteroides* and better metabolic-inflammatory responses [36]. Other cofactors such as pre-pregnancy BMI (p-BMI) affected microbiome profiles through interacting with diet and influenced GDM status [37]. Negligible differences were found among obese pregnancies suffering from GDM or not, and the microbiota was more stable and limited the capacity to respond to the diet [38]. *Akkermansia* enrichment only appeared in non-obese GDM after caloric control [38]. Additional 5 out of 17 differential abundance were found in GDM group after adjusting for p-BMI [14]. The sustained and heterogeneity of IR influenced microbiota diversity through effects on metabolic profiles. It showed that higher IR level was associated with lower microbial diversity adjusting for BMI or not [40]. Glucose level and gestational weight gain (GWG) were also essential, manifested as dominant *WAL 1855D* and *Bacteroidetes* by hierarchical clustering in excessive GWG group [41–43]. Hence, it is conducive to take these cofactors into account for better understanding changes of microbiota composition suffering from GDM.

To date, previous studies reported a unique microbiome pattern in GDM. Most studies examined at single time adopting 16 S rRNA sequencing without adjusting for confounding factors, and thus there are still controversies concerning associations between gut microbiome and GDM. Although GDM status was main in affecting microbiome changes, there are several factors that can influence the studies on gut microbiota of women with GDM. The physiological factors include different diagnostic criteria and demographic characteristics such as gestational age, diet habit, glucose control level, antibiotic use, p-BMI, GWG and IR level as mentioned above. The experimental factors mainly include sampling and DNA extraction method, preservation condition, and sequencing platform. After controlling the potential influencing factors, it could be more objective to reflect the relationships between microbiome characteristics and GDM development. Dynamic changes were also needed. For example, the temporal increase of microbiome-derived propionate from T1 to T2 was greater in control group [18], indicating a strong competence against glucose intolerance, although there were no significant differences at each trimester. The introduction of new methods (such as MLGs, CMRS et al.) and combination analysis (such as metabolomics) are more helpful in understanding correlations between the flora and environment.

## The possible implications of microbiome derived SCFAs in pregnancy and GDM

SCFAs are synthesized from gut microbiota through fermentation of non-digestible fibers, proteins and glycoproteins. Acetate, propionate and butyrate constituted > 95% of SCFA contents, and the proportion of each is appropriately 60:20:20. The bacteria responsible for acetate production is widely distributed, whereas the production pathways for propionate and butyrate appear highly conserved and substrate specific [44]. Propionate is produced through succinate, acrylate or propanediol pathway from *Bacterioidetes* and some *Firmicutes* (*Veillonlla*, *Megasphera* et al.) [45]. Specific families belonging to *Clostridiales* produced butyrate through butyryl-CoA, phosphotransbutyrykase and butyrate pathways [45, 46].

The roles of SCFA have been identified during normal pregnancy, GDM, obesity and multiple sclerosis. They were closely related with metabolic parameters and provide evidence for intervention potential. During normal pregnancy, circulating propionate was negatively associated with leptin, infant length and body weight [8]. Butyrate in human milk was inversely associated with infant weight and BMI at 3 and 12 months, offering beneficial effects in weight gain and adiposity [47]. Caesarean section (CS)-delivered infants uniquely produced excess butyrate through enriched bacteria, and provided novel insights between delivery mode and infant health [48]. Acetic, propionic and butyric acid were all positively correlated with total cholesterol (TCHO), high density lipoprotein (HDL) and triglycerides (TG). HDL was only positively related with propionate in overweight/obese pregnancies [49]. As for anthropometric parameters and carbohydrate metabolism, the three dominant SCFAs were positively related with p-BMI, HbA1c contents, glucose value at three OGTT timepoints, and inversely related with body weight gain and insulin level [50]. GDM pregnancies are more capable of oxidizing sugars than lipids and characterized by IR and low-grade inflammation. In GDM, propionate was positively correlated with insulin in T2 and maintained until T3 [51]. The butyrate was negatively correlated with WBC counts, neutrophil counts, p-BMI, GWG per week before GDM diagnosis, and ponderal index, but positively correlated with TCHO and LDL levels in all pregnancies [51]. Another study found no relationships between main SCFAs and clinical parameters in GDM [52]. This could be explained in terms of their roles as energetic substrates or signaling molecules, and will be discussed below.

These observations were largely relied on measurements of stool or circulating SCFAs. However, it is still unclear whether the stool SCFA output was suitable to represent luminal production. Circulating SCFA contents were more representative since approximately 95% of colonic SCFAs were absorbed into blood and connected with metabolic health [53].

## Mechanisms of microbiome-host interactions in GDM and metabolic disorders through SCFAs

### SCFAs exerted metabolic effects as energy sources in local and peripheral tissues

SCFAs are important mediators between gut microbiome and host. Butyrate constituted 60–70% energy source for epithelial cells through β-oxidation. It promoted intestinal epithelial cell growth and enhanced gut barrier integrity, thus impeded bacteria from gut lumen for entering the circulation and avoided GDM onset [54]. Butyrate and acetate were direct substrates for cholesterol and fatty acids synthesis, and induced decrease in lipolysis and improved IR in liver and adipose tissue [55]. They also reduced lipid accumulation in an AMPK-dependent manner. Butyrate was converted to butyryl-CoA and further increased CPT1A activity, accelerated fatty acid oxidation (FAO) and promoted inducible regulatory T cell differentiation for maintenance of immune-metabolic homeostasis [56]. Acetate also exerted anti-lipolytic effect by reducing free fatty acid flux to the liver and attenuated fatty liver induced deterioration in glucose intolerance [57]. The propionate was precursor for glucose synthesis in liver. The intestinal gluconeogenesis promoted glucose release in portal vein, which resulted in decreased hepatic glucose production and increased energy expenditure through a brain-related mechanism [58]. The beneficial effects of diet enriched in propionate and butyrate were abolished in mice deficient in intestinal gluconeogenesis. Daily propionate supplementation was associated with decreased 2 h post-prandial glucose level due to decreased digestion of bread-derived starch. It was speculated that SCFAs entered an appropriate point of the Krebs cycle and mitigated the need for glucose as the sole energy substrate [59]. A high-fiber diet significantly increased key enzymes production of acetate (formate-tetrahydrofolate ligase) and butyrate (butyryl-coenzyme A) in T2DM, and stimulated GLP-1 secretion [60]. More evidence is needed on the role of butyrate alone or combined with propionate in energy regulation. There were few studies on SCFA as energy sources in GDM. Considering the characteristics of metabolic disorders, it is possible that similar mechanisms are preserved in GDM pregnancies when imbalance between energy storage and release occurred.

SCFAs have biphasic effects on energy control due to different types and concentrations. In humans, there is a strong biological gradient from production site to downstream tissues. In the proximal part of colon with increased availability of carbohydrates and water, the concentration is nearly 70-140mM, and decreased to 20-70mM in the distal part [61]. The output of splanchnic propionate or butyrate reached millimolar concentrations given that the hepatic SCFA utilization balanced its production under physiological status [62]. In the blood, the levels are ranged from 100–200µM for acetate, and 1–20µM for propionate and butyrate [63]. The overflow of SCFAs caused adverse effects due to lipogenic effect and energy accumulation. In TLR5-deficient mice, the overgrowth microbiome was accompanied by elevated SCFA levels, leading to increased hepatic de novo lipogenesis and metabolic impairment [64]. Totally, it is important to understand their biological effects to achieve clinical translation.

### The potential roles of SCFA as signaling molecules through GPCRs and/or HDACs

#### GPCRs

**Fig. 2:**
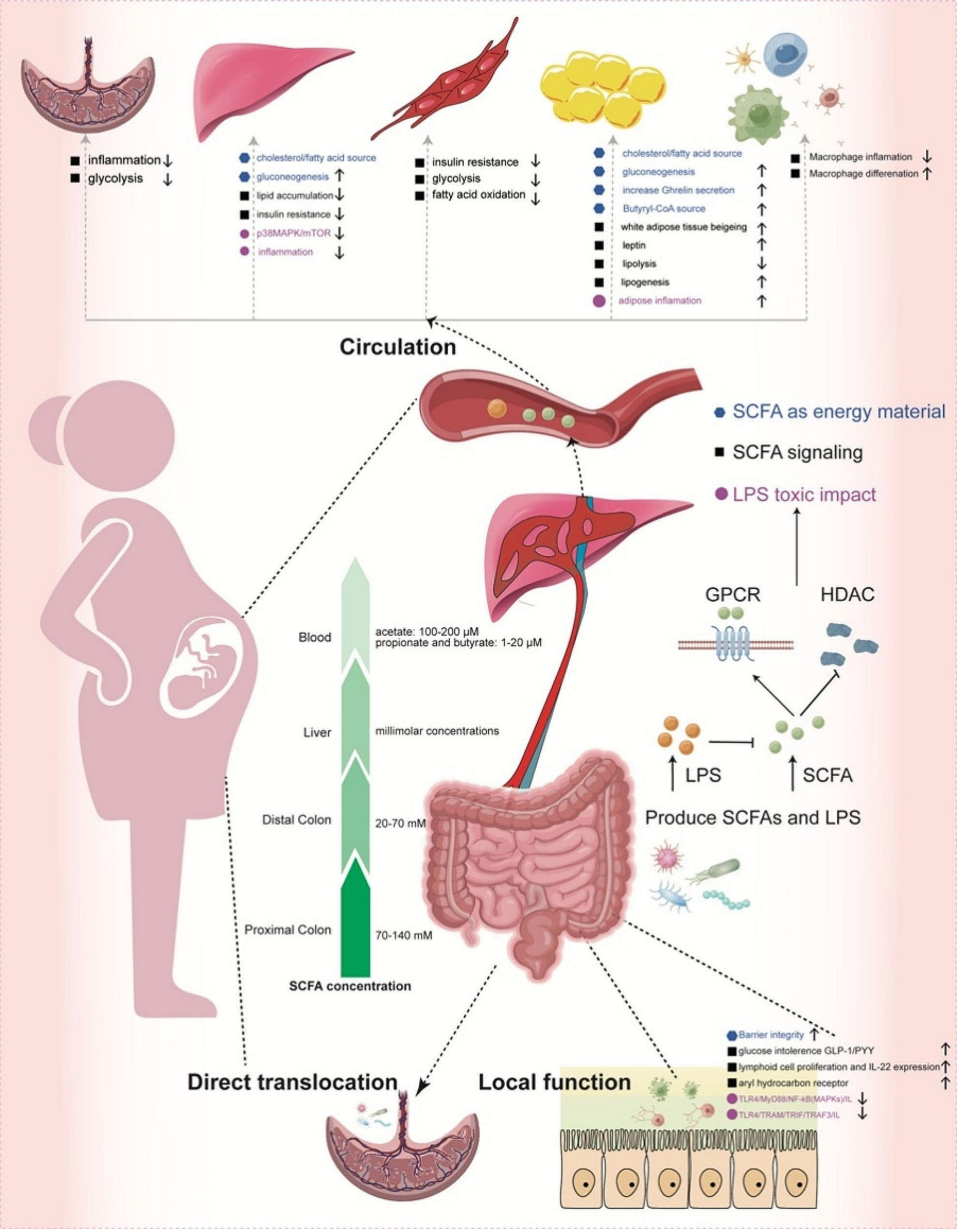
The direct or indirect roles of microbiome on GDM and metabolic disorder development. In metabolic disorders, the SCFA functioned as energy sources (blue) or signaling molecules through GPCRs and/or HDACs (black) in local intestinal tract and periapical tissues. The gut microbiome influenced placental structure and functions through direct translocation or SCFAs, which established gut-placenta axis. The LPS overproduction is another characteristic of gut microbiota dysbiosis (pink). LPS alone or in combination with SCFA reduction involved in the occurrence and development of GDM

SCFAs acted locally or systemically as signaling molecules through coupling with G-protein coupled receptors (GPCRs) or histone deacetylases (HDACs) (Fig. 2). GPCR41/43 were most important and ubiquitously expressed in intestinal epithelial cells (IEC), liver, and gestational tissues [65]. Previous data suggested that SCFAs promoted GLP-1 and PYY release in enteric L cells through GPCR41/43 directly. Mice lacking them exhibited glucose tolerance impairment [66]. GPCR43 regulated innate lymphoid cell proliferation and IL-22 expression via Akt-Stat3 axis, and afforded protection from intestinal inflammation [67]. Butyrate also promoted IL-22 production through aryl hydrocarbon receptor (AHR) and HIF-1α in GPCR41/HDAC dependent manner [68]. The antimicrobial peptides (AMPs) are important components produced by IEC in maintaining immune homeostasis. SCFA supplement induced RegIIIγ and β-defensins, and avoided IgA responses in wild type but not in *GPCR43*^*−/−*^ mice by activating mTOR and Stat3 [69, 70].

The impacts of SCFAs go beyond local cells. In white adipose tissue (WAT) of DHA/EPA-treated *db/db* mice, the GPCR41/43 and beigeing program markers (PRDM16, PPARγ) were upregulated, accompanied by elevated propionate, butyrate, *Bifidobacterium* and *Lactobacillus*, and improved glucose status [71]. It is supplied that DHA/EPA enhanced SCFA generation and WAT beigeing through GPCRs, linked gut to adipose and established the gut-organ axis. High-fiber diet was less likely to develop diabetic nephropathy with improved microbiome structure and elevated SCFA concentrations. The protective effects were disappeared in mice lacking genes encoding GPCR43/109A [72]. GPCRs functioned by binding to different subtypes. In human renal cortical epithelial cells, propionate elicited inhibitory effects by phosphorylation of p38MAPK and JNK through Gβγ (i/o) subtype [73]. However, GPCR43 contributed to inflammasome activation and played pathogenic roles in macrophages [74]. Therefore, GPCR43 exerted dual effects depending on cell types and locations in peripheral tissues. For normal labor, the elevated receptors are essential through dampening down pro-inflammatory responses [75]. *GPCR43*^*−/−*^ pregnant mice developed fasting hyperglycemia, diminished β-cells expansion and decreased circulating propionate, explaining microbiome contributions on gestational glucose homeostasis [76]. In high-fat diet (HFD) rats before and during gestation, the contents of propionate, GPCR43 and placental labyrinth zone thickness were significantly decreased which destroyed nutrient provision and heightened inflammation through propionate-GPCR43 axis [77]. However, 5mM butyrate and 10mM propionate incubating for 1 h significantly suppressed expressions of pro-inflammatory cytokines, chemokines in placental explants through ERK activation independent of GPCR [78]. In primary human cells isolated from myometrium and fetal membranes, preincubation 5mM butyrate and 20mM propionate for 1 h increased adhesion molecules in GPCR independent manner [79]. Considering the double effects of SCFA-receptor interactions, more experiments are warranted.

#### HDACs

HDACs played essential roles in modifying chromosomal structure and gene expression [65]. Butyrate strongly inhibited HDAC activity and upregulated IL-10 with 0.5mM for 48 h through MAPK signaling for immunological tolerance maintenance in B10 cells [80]. In IECs, butyrate repressed indoleamine 2,3-dioxygenase 1 (IDO1) expression in dose-dependent manner ranging from 0.5 to 8mM for 24 h [81]. In neutrophils and bovine mammary epithelial cells, the propionate (≥ 4mM) and butyrate (≥ 0.4mM) reduced TNF-α and CINC-2αβ production, and higher concentrations (12mM for propionate and 1.6mM for butyrate) inhibited NO and cytokines production [82]. However, 4mM butyrate and propionate induced neutrophil chemotaxis in a time-dependent manner over 20 h only through inhibiting class I and II HDACs [83]. The HDAC 2 and 8 was mostly inhibited by propionate and butyrate, and HDAC3 was additionally inactivated by butyrate [84]. This demonstrated that butyrate and propionate induced proliferation or apoptosis through specific HDAC depending on the concentrations and cell types. “Butyrate paradox” also contributed which promoted cell proliferation cultured in normal medium by functioning as oxidative energy sources, while inhibited cells cultured in high-glucose medium as HDAC inhibitors being metabolized at relatively low levels [85].

In human umbilical vein endothelial cells, the anti-inflammatory effects of SCFAs were facilitated by simultaneously activating GPCRs and inhibiting HDACs [86]. Through activating GPCRs, IL-6 was significantly reduced by pre-incubation with 10mM acetate for 16 h, 0.3mM propionate and 0.1mM butyrate for 24 h. IL-8 was obviously decreased by acetate. HDAC activity was inhibited in the condition of 0.1mM butyrate and 0.3mM propionate with 12 h treatment, 5mM butyrate with 6 h treatment, or 10mM propionate after 48 h treatment. This indicated that whether SCFA acted as energetic substrates or signaling molecules depended on its concentration and target tissue. Summarily, most experiments were carried out using nontoxic SCFA concentrations found in the intestinal tract, which are higher than those in the blood. More studies are warranted to investigate the bilateral effects on trophoblast-derived cells and the associations with gestational complications. In addition to “indirect” associations between gut microbiome and placenta, the biological plausibility has also involved the immediate translocation of gut pathogens to invade the fetal-placental unit, which provided new perspective on microbiome-placenta axis in disease development [87, 88].

### LPS-induced low grade inflammation and gut permeability

**Fig. 3:**
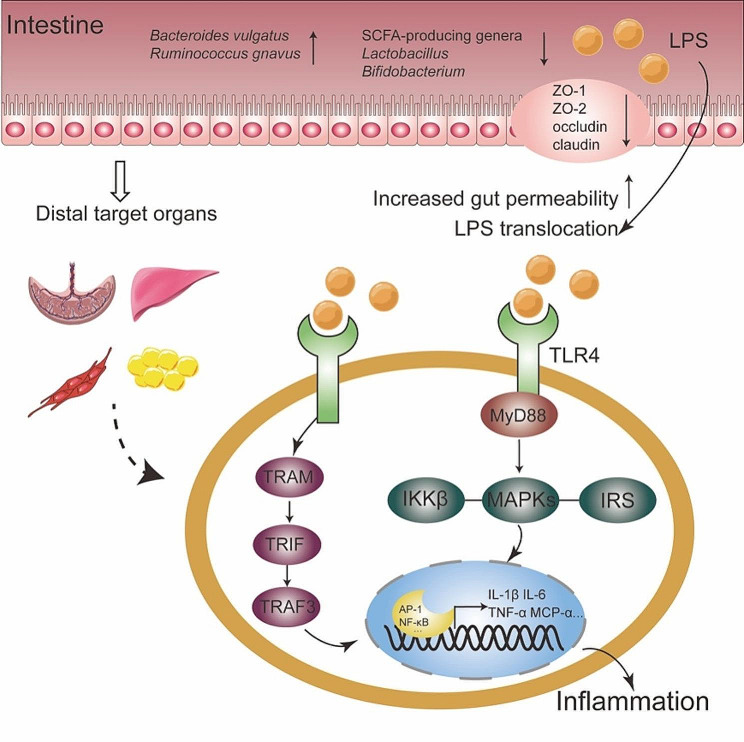
The effects of LPS and inflammation on microbiome in GDM. LPS overproduction is one of characteristics of gut microbiota dysbiosis in GDM. It resulted in reduced expression of tight junction proteins (TJP) which led to abnormal gut permeability and LPS translocation. This further influenced inflammatory responses on distal organs and whole body in MyD88 dependent and independent manner

LPS overproduction is one of characteristics of gut microbiota dysbiosis, which induced inflammatory responses and diabetes development (Fig. 3). It resulted in reduced expression of tight junction proteins (TJP) including zonula occludens-1 (ZO-1), claudin and occludin. The breakdown of tight junction function led to abnormal gut permeability and LPS translocation [89]. *Bacteroides vulgatus* and *Ruminococcus gnavus* were significantly positively correlated with LPS biosynthesis in GDM [29]. Subsequently, LPS initiated inflammation via TLR-mediated MyD88-dependent pathway and transcription of IL-6 and TNF-α. It also stimulated inflammatory mediators in MyD88-independent signaling through TLR4-TRAM-TRIF-TRAF3 cascades [90, 91]. In HFD rats characterized by enhanced IR, fecal LPS level was increased parallel with upregulated MCP-α and IL1-β in plasma. The SCFA-producing genera *Bacteroidetes*, *Prevotella spp.* and *Lactobacillus spp.* were decreased in HFD group [92]. LPS stimulation obviously upregulated the expression of GPCR41/43 and proinflammatory cytokines, and were attenuated when incubating with 20mM acetate or propionate for 8 and 24 h [93]. Mitochondrial antiviral signaling protein (MAVS) is a component of innate immunity to maintain intestinal integrity. LPS administration accelerated injuries in MAVS knockout diabetic mice and showed more severe kidney injuries and elevated IL-17 expression [94]. The gut barrier dysfunction, decreased SCFA concentration and activated TLR4/NF-κB pathway were reversed by chemical compounds [95, 96], and blocked in intestinal flora deficient mice [97]. It proved that SCFAs were important regulators of TJP to protect barrier integrity, inhibit LPS-stimulated inflammation, attenuate oxidative stress and improve metabolic parameters through GPCRs and/or HDACs.

## The effects of GDM status and therapy on gut microbiota and metabolism in offspring and potential mechanisms

### The characteristics of gut microbiota and metabolism in GDM infants and transmission mechanisms

The exposure to prenatal metabolic stress contributed to health outcomes in offspring. The vertical transmission of maternal gut microbiome triggered metabolic disease later in life, particularly from pregnancy to 1–2 years after birth [11, 98]. The infants born from GDM mothers showed decreased α and β-diversity in meconium, higher abundance of pro-inflammatory taxa including *Escherichia*, *Parabacteroides* and *Bacteroides*, and decreased *Prevotella* and *Lactobacillus* [99, 100]. The genus *Bacteroides* was related to type 1 diabetes (T1DM) development [101]. The genus *Prevotella* is a taxonomic biomarker of normal gestational glucose control and associated with higher insulin sensitivity [102]. The microbial co-occurrence network showed balanced correlations and less disrupted ecology in control group, while genus *Rothia* and *Clostridium sensustricto* were increased in infants born to women with GDM which caused infection and metabolic disease of childhood [103]. The disturbed microbiota also contributed to increased BMI Z-score at 12 months of age which suggested risks of childhood obesity [99, 104]. The oral *Veillonella* was associated with periodontal diseases and was depleted in gut microbiome among offspring born to GDM during first week of life and 9 months later [102, 104]. In a study followed up until 5 years postpartum, genera *Anaerotruncus* and *Victivallis* were all more abundant in children of GDM women, and the former was positively related with glucose intolerance and gut permeability [105]. These results suggested that microbiome variations during early life could be sustained and may be associated with abnormal glucose metabolism in later life. The β-diversity and flora constitution were more similar between mother and her own child varying by the same trend when compared with unrelated children, revealing the intergenerational concordance associated with GDM [106, 107]. Maternal diet also influenced infant microbiome colonization. The infants born to mothers with CHOICE or higher-complex carbohydrate diet exhibited greater *Clostridiaceae* and *Bifidobacterial*, and decreased *Enterococcaceae*. The reduced opportunistic pathogens were incapable of activating immune-metabolic responses [107, 108]. In animal studies, the *Lactobacillus spp.* was significantly lower and the potentially pathogenic flora such as *E. coli* was more abundant in HFD offspring, and contributed to higher serum glucose level, TG and HDL level until adulthood [109]. Low-calorie sweeteners addition during pregnancy exaggerated gut microbiota dysbiosis and directly influence glucose intolerance at weaning of offspring through FMT experiments [110]. Recent study found that the species *Lactobacillus* mentioned above and its metabolite phenyllactic acid (PLA) limited HFD-induced obesity during early life in a peroxisome proliferator-activated receptor γ (PPAR-γ) dependent manner in IECs [111]. Maternal and post-weaning high fat diet also led to higher fasting glucose and HOMA-IR level and decreased *Bacteroidetes* abundance at 32 weeks of age in offspring, which was obviously associated with glucose response to the glucose load [112]. In conclusion, current studies showed that gut microbiota profile was disturbed in GDM offspring and influenced metabolic status. More animal researches are warrant to provide direct evidence to explore the mechanism of early life microbial changes on metabolism later in life.

For mechanism study, the dysbiosis in GDM continued and influenced offspring through FMT experiments, manifested as increased *Oscillibacter* and depleted *Akkermansia*, *Parvibacter* and SCFA contents [113, 114]. In obese mice induced by HFD, the reshaped maternal gut microbiome and decreased placental GPCR43 resulted in lipid dysmetabolism of fetal liver and reprogramming [77]. Propionate promoted embryonic sympathetic neuronal and enteroendocrine differentiation directly through GPCR41/43, and improved obesity resistance [115]. In maternal low-fiber diet model, butyrate supplement improved cognitive function and synaptic plasticity in offspring through inhibiting HDAC4 [116]. The mother-to-neonate microbiota transmission was influenced by several factors such as nutritional habits, delivery mode and breastfeeding [100, 117, 118]. Overall, more direct evidence was needed to explore the mechanism by which GDM mothers influence their offspring in gut microbiome dependent pathway.

### The effects of GDM therapy on gut microbiota and metabolism in offspring

Gut microbiota had become intervention target for GDM given its important roles in disease development. Combined nutritional and exercise therapy are first-line treatments and exert profound effects on GDM pregnancies and offspring [119, 120]. The pharmacological intervention is added if optimal glucose levels are not obtained. The effects of insulin on gut microbiota has not been extensively studied. One study found that the proportion of *Clostridiales*, *Lactobacillus* and *Bacteroidetes* were higher in women accepting insulin treatment and could be transferred to newborns [121]. In a hyperglycemic mouse model induced by HFD, *Bacteroidtes* was obviously downregulated and *Firmicutes, Deferribacteres* and *Actinobacteria* were increased after insulin therapy [122]. In addition to insulin, optional agents such as metformin, probiotics, prebiotics and synbiotics have been gradually promoted due to their regulatory effects on gut flora and metabolism in humans. The effectiveness and safety have been verified through clinical and animal studies. Here, we mainly focus on their effects on gut flora and metabolism in offspring.

#### Metformin

Metformin is considered to change intestinal microbiota profiles and improve metabolic problems. Most clinical studies were performed among patients suffering from T2DM. One study aimed to elucidate differences in maternal microbiota composition and function in GDM treated with metformin or insulin. It was showed that genus *Firmicutes* and *Peptostreptococcaceae* were declined while *Proteobacteria* and *Enterobacteriaceae* were increased with metformin therapy [123]. The enriched members were inversely correlated with maternal mean postprandial glycemia and gestational weight gain. Further analysis with a large sample size adopting metagenome/transcriptomics and follow-up to offspring is encouraged. In an animal experiment, metformin significantly reduced maternal *Verrucomicrobia* abundance and upregulated claudin-3 level induced by HFD [124]. In the fetal intestine, the level of pro-inflammatory marker IL-6 and apoptotic cells were also obviously inhibited [124]. Another study found that the expression levels of other TJPs such as ZO-1, occludin and claudin-4 were restored in adult male offspring after maternal metformin treatment [125]. The genera *Clostridium* and *Lactobacillus* were both enriched, and improved the body fat composition in themselves and the offspring [125].

#### Probiotics, prebiotics and synbiotics

Probiotic is defined as live microorganism which when administered in adequate amounts confers a health benefit on the host [126]. Prebiotics are selectively used by host microorganisms particularly *Lactobacilli* and *Bifidobacterium* and can be found in wheat, bananas and onion [126, 127]. The synbiotic is commonly composed of a probiotic combined with a prebiotic, and target on autochthonous microorganisms [126]. Single or multiple strains of probiotics supplementation during normal pregnancy exerted beneficial effects on infant microbiome and metabolism [128]. According to RCT related to GDM, the probiotics and/or fish oil intervention did not prevent GDM in overweight and obese women [129, 130]. However, they exhibited therapeutic effects and controlled glucose and lipid metabolism in women when GDM occurred [131, 132]. The effects did not transfer to neonates and influence their body weight or immune system [132, 133]. More clinical studies focus on infant microbiome and metabolism born to GDM were needed. In HFD animal model, maternal probiotics intervention ameliorated fecal microbiota dysbiosis at weaning, and the male pups was more susceptible [134]. Among adult pups, the glucose and insulin levels were decreased only in female pups accompanied by increased *Bacteroidetes S24-7*, which was negative correlated with glucose level [134]. The sex-dependent effect may be linked to sex hormones and the underling mechanism is unclear. In the pig offspring, maternal probiotics and synbiotics supplementation also increased the abundance of several beneficial bacteria such as *Actinobacteria, Clostridium, Gemmiger, Blautia, and Roseburia*. The colonic acetate and butyrate concentrations were simultaneously increased [135]. The prebiotics such as polydextrose and oligo-fructose was associated with a better metabolic status as presented by a strong clearance of glucose especially in female offspring and lower LPS level. They also increased abundance of *Bacteroides* and *Bifidobacterium spp* at early stage [136, 137]. However, some studies found that maternal supplementation of probiotics and prebiotics with HFD exerted few and even harmful effects on offspring microbiome and metabolism [138–140]. This suggested that the type, amount, treatment period of these agents and the physiological conditions were essential in influencing the results.

## The epigenetic links between GDM and gut microbiome and their effects on offspring metabolism

The basic epigenetic signals including DNA methylation, histone modifications, noncoding RNA regulation and chromatin remodeling were considered to be involved in GDM pathophysiology. Fetal development could be further influenced by regulating genes required for the epigenomic reprogramming process in the utero. The altered microbiota seemed to be one of the most important participants of this process [141]. The microbiome derived SCFAs were widely accepted as substances in epigenetic regulation through targeting on GPCRs and/or HDACs discussed above, which served as a link between maternal microbiome and fetal health in GDM. Butyrate also regulated DNA methylation by downregulating DNMT1, demethylating downstream genes such as p21 [142]. Besides, the SCFA-producing genera *Bifidobacterium spp* and *Roseburia* exerted anti-inflammatory effects by reducing DNA methylation TRIB1 gene-mediated COX-2 expression and upregulating PGC1𝛼 gene respectively in neonatal diabetes [143]. Metformin crossed the placenta freely and had epigenetic effects on fetus via AMPK signaling [144]. Other microbiota synthesized metabolites such as biotin, folate and betaine were also involved in chromatin remodeling by modifying histones or in 5-methyltetrahydrofolate metabolism [142]. In conclusion, gut microbiota may involve in GDM pathology in an epigenetic dependent manner through its metabolites, which can be vertically transmitted to their offspring [142, 145]. Any factors influencing microbiome composition including epigenetics diets, probiotics/prebiotics and metformin, could change gene levels involved in epigenetic and posttranscriptional regulation [145]. This provided new perspectives for GDM intervention mechanism.

## Conclusion

Based on previous studies, the gut microbiome and derived SCFAs involved in GDM initiation and development, and further exerted influences on their offspring. SCFAs had strong ability in regulating immune-metabolic responses, while the underlying mechanisms remained unclear. The epigenetic regulation may be essential. The SCFA-coupled GPCRs and HDACs were ubiquitously expressed in gestational and embryonic tissues. Depending on concentrations and cell types, SCFAs bound to different downstream molecules and involved in specific physiological processes, which laid the foundation of microbiota-placenta or microbiota-fetus axis establishment. Recent FMT studies showed that the microbiome influenced placental structure and development, especially nutrient transport functions through SCFAs [146, 147]. Multi-omics approach reveled close relationships between host metabolomes in evaluating risks of neonatal inborn errors of metabolism, providing new evidence of effects of maternal gut flora on offspring. However, few studies have analyzed the relationship between gut microbiota and GDM development through the genetics, metabolomics and gut microbiota. It is needed to determine differences between normal and GDM pregnancies, and their concordance variations with offspring. Based on these, the gut microbiota interventions might become novel technology to reduce GDM risk, the GDM-induced complication risks and childhood metabolic disorders.

## Data Availability

No datasets were generated or analysed during the current study.
